# Diagnostic Performance of F-18 FDG PET/CT Compared with CA125, HE4, and ROMA for Epithelial Ovarian Cancer

**DOI:** 10.31557/APJCP.2021.22.4.1123

**Published:** 2021-04

**Authors:** Sun Seong Lee, Ji Sun Park, Kyung Bok Lee, Dae Hoon Jeong, Jung Mi Byun, Seok Mo Lee

**Affiliations:** 1 *Department of Nuclear Medicine, Busan Paik Hospital, University of Inje College of Medicine, Busan, Republic of Korea. *; 2 *Department of Obstetrics & Gynecology, Busan Paik Hospital, University of Inje College of Medicine, Busan, Republic of Korea. *; 3 *Department of Nuclear Medicine, Busan Seongso Hospital, Busan, Republic of Korea. *

**Keywords:** F-18 FDG PET/CT, epithelial ovarian cancer, ROMA score, SUVmax

## Abstract

**Objective:**

This study aimed to examine the diagnostic performance of F-18 fluorodeoxyglucose positron emission tomography with computed tomography (F-18 FDG PET/CT) compared with cancer antigen 125 (CA125), human epididymis protein 4 (HE4), and risk of ovarian malignancy algorithm (ROMA) score to distinguish epithelial ovarian cancer from benign tumors.

**Methods:**

A total of 46 patients with pelvic masses, who underwent F-18 FDG PET/CT, CA125, and HE4 before surgery between January 2015 and December 2018, were included in this retrospective study. The diagnostic performance of CA125, HE4, ROMA score, and maximum standardized uptake value (SUVmax) to differentiate epithelial ovarian cancer from benign pelvic tumors was examined by receiver operating characteristic curve analysis.

**Results:**

Among the 46 patients, 28 were cases of ovarian cancers and 18 were of benign. The mean values of CA125, HE4, ROMA score, and SUVmax were significantly higher in the ovarian cancer group than the benign group. In early cancer stages (stages I and II), Area under the curve for SUVmax was significantly higher than CA125 and ROMA score (0.778 for CA125, 0.753 for HE4, 0.682 for ROMA score, and 0.922 for SUVmax).

**Conclusion:**

SUVmax using F-18 FDG PET/CT showed a high diagnostic accuracy for differentiating epithelial ovarian cancer from benign pelvic tumors, including early stage ovarian cancer. F-18 FDG PET/CT can be a useful modality for the assessment of pelvic mass.

## Introduction

Approximately 21,000 new ovarian cancer patients have been diagnosed, and 14,000 deaths due to ovarian cancer occurred every year in the United States in 2020. Despite the low incidence rate of ovarian cancer, the mortality rates are high, therefore, early diagnosis is crucial (Siegel et al., 2020). However, early diagnosis is difficult because ovarian cancers often exist with no specific symptoms; approximately only 30% of the patients are diagnosed at an early stage. Of the 5-year survival period, approximately 90% is achievable for patients with stage I disease, but only 20% is achievable for patients in the stage IV disease with an appropriate treatment (Torre et al., 2018). Therefore, an early detection is necessary to increase the cure rate and decrease the mortality rate.

It is often difficult to differentiate malignant ovarian tumors from the benign ovarian tumors before surgery. Cancer antigen 125 (CA125) and human epididymis protein 4 (HE4) have been used as tumor markers for diagnosing ovarian cancer and predicting prognosis (Steffensen et al., 2011; Furrer et al., 2019). However, serum CA125 levels can be elevated in various benign conditions, such as endometriosis and pelvic inflammatory disease. The level can change during the menstrual cycle, resulting in low specificity (Meden and Fattahi-Meibodi, 1998; Moore et al., 2012). Serum HE4 is not dependent on the menstrual cycle, and HE4 has shown a better specificity as compared to CA125 (Hallamaa et al., 2012). Recently, the risk of ovarian malignancy algorithm (ROMA) score, a combination of CA125, HE4, and the menopausal status of patients, has been used for diagnosing and assessing the prognosis of ovarian cancer with a high sensitivity and specificity (Moore et al., 2009).

F-18 fluorodeoxyglucose positron emission tomography with computed tomography (F-18 FDG PET/CT), which evaluates glucose metabolism in tumors, is widely used for diagnosis, tumor staging, and therapy monitoring. Moreover, in ovarian cancer, the usefulness of F-18 FDG PET/CT for differential diagnosis, staging, and therapy monitoring is well known (Zytoon et al., 2013; Rubello et al., 2018; Kemppainen et al., 2019).

To the best of our knowledge, the diagnostic performances of F-18 FDG PET/CT and ROMA score have not been compared to date. Therefore, to examine the diagnostic performance of F-18 FDG PET/CT compared with CA125, HE4, and ROMA score to distinguish epithelial ovarian cancer from benign tumors.

## Materials and Methods

*Patients*

A total of 46 patients with pelvic masses, who underwent F-18 FDG PET/CT, CA125, and HE4 before surgery between January 2015 and December 2018, were included in this retrospective study. All patients underwent resection of pelvic masses and were categorized into benign or epithelial ovarian cancer groups by histopathological diagnosis. The following patients were excluded from the study: 1) patients with a history of cancer or with any other type of cancer and 2) patients under 18 years of age.

*FDG PET/CT imaging*

All patients enrolled in the study fasted for at least 6 h before the PET/CT scan. The blood glucose levels of the patients were checked before the injection of F-18 FDG and did not exceed 200 mg/dl. A whole-body scan from head to upper thigh was acquired 60 min post intravenous injection of approximately 370 MBq of F-18 FDG. PET/CT examinations were performed using a PET/CT scanner (Discovery STE; GE Healthcare, Milwaukee, WI, USA). The CT images were obtained with a reference of 140 kV, 60–80 mA, and a section thickness of 3.75 mm. Then PET data were acquired for 2 min per bed position. PET images were reconstructed using an ordered-subset expectation maximization iterative reconstruction algorithm and then fused with the CT images.

*Image evaluation*

PET/CT images were reviewed by an experienced nuclear medicine physician who was aware of each patient’s clinical history. For the semi-quantitative analysis, three-dimensional region of interest was drawn on the primary tumor to calculate the maximum standardized uptake value (SUVmax). Clinical stages were assigned according to the American Joint Committee on Cancer manual, 8^th^ edition.

*Histopathological analysis*

Histopathological analysis was conducted on the representative sections of surgical specimens from patients. The tumors were classified as epithelial ovarian cancer, non-epithelial ovarian cancer, and benign tumor based on the histopathological characteristics.

Blood samples were obtained within 4 weeks prior to PET/CT and surgery. Serum CA125 concentrations were assessed with the electrochemiluminescence (ECLIA) technique on a Cobas e801 (Roche Diagnostics, Switzerland) analyzer, and HE4 concentrations were assessed with the ECLIA technique on a Modular PE analyzer (Roche Diagnostics, Switzerland) according to the manufacturer’s instructions.

*ROMA score calculation*

The ROMA score was calculated using the following algorithm. (Moore et al., 2009)

Premenopausal: PI (predictive index) = −12.0 + 2.38 * In (HE4) + 0.0626 * In(CA125)

Postmenopausal: PI (predictive index) = −8.09 + 1.04 * In (HE4) + 0.732 * In(CA125)

ROMA score = exp (PI) / [1 + exp (PI)] * 100

*Statistical analysis*

Statistical analyses were performed using MedCalc for Windows, version 19.6 (MedCalc Software, Ostend, Belgium). The Mann-Whitney test was used to compare the mean values of CA125, HE4, ROMA score, and SUVmax between malignant and benign groups. Receiver operating characteristic (ROC) curve analysis was performed to examine the diagnostic performance of CA125, HE4, ROMA score, and SUVmax to differentiate epithelial ovarian cancer from benign pelvic tumors. Sensitivity, specificity, and area under the curve (AUC) were calculated. For all statistical comparisons, a P value less than 0.05 was considered significant.

## Results

The mean age of patients was 53.9 ± 15.3 years. Among the 46 patients, 28 were cases of ovarian cancers and 18 were of benign; 20 were premenopausal, and 26 were postmenopausal. Among the 28 cancer patients, 11 were at stage I-II, and 17 were at stage III-IV. In the benign group, mean CA125, HE4, ROMA score, and SUVmax were 45.7 ± 67.3, 54.5 ± 21.4, 17.1 ± 16.6, and 2.8 ± 1.7, respectively. In the ovarian cancer group, mean CA125, HE4, ROMA score, and SUVmax were 659.5 ± 1114.2, 432.0 ± 473.2, 64.3 ± 36.2, and 11.2 ± 4.4. There were significant differences in CA125, HE4, ROMA score, and SUVmax between the benign and ovarian cancer groups (Table 1).

**Figure 1:**
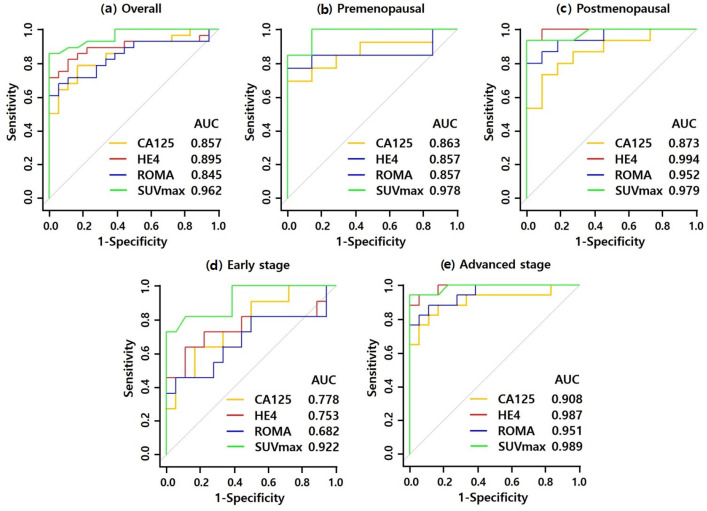
ROC Curves of CA125, HE4, ROMA Score, and SUVmax in Distinguishing Ovarian Cancers from benign Tumors. (a) ROC curves of all patients. (b) ROC curves of premenopausal patients. (c) ROC curves of postmenopausal patients. (d) ROC curves of early stage. (e) ROC curves of advanced stage. AUC, area under curve; CA125, Cancer antigen 125; HE4, human epididymis protein 4; ROMA, risk of ovarian malignancy algorithm; SUVmax, maximum standardized uptake value; ROC, receiver operating characteristic curve

In ROC curve analysis, the AUC for distinguishing ovarian cancer from benign pelvic tumor was 0.857 for CA125, 0.895 for HE4, 0.845 for ROMA score, and 0.962 for SUVmax. There were significant differences between the CA125 and SUVmax, and ROMA score and SUVmax (Figure 1a).

Subgroup analysis was conducted according to the menopausal status: In the premenopausal group, the AUC was 0.868 for CA125, 0.857 for HE4, 0.857 for ROMA score, and 0.978 for SUVmax. There were no significant differences between the values (Figure 1b). In the postmenopausal group, the AUC was 0.873 for CA125, 0.994 for HE4, 0.952 for ROMA score, and 0.979 for SUVmax. There were significant differences only between the CA125 and ROMA score. Otherwise, there were no significant differences between the values (Figure 1c).

Subgroup analysis was conducted according to the cancer stage: In early cancer stages (stages I and II), AUC was 0.778 for CA125, 0.753 for HE4, 0.682 for ROMA score, and 0.922 for SUVmax. There were significant differences between the CA125 and SUVmax, and ROMA score and SUVmax (Figure 1d). In advanced disease stages (stages III and IV), the AUC was 0.908 for CA125, 0.987 for HE4, 0.951 for ROMA score, and 0.989 for SUVmax. There were no significant differences between the values (Figure 1e).

The best cut-off value of CA125 for the differentiation of ovarian cancer from benign tumors was 46.15, with a sensitivity of 78.6% and a specificity of 83.3%. The best cut-off value of HE4 was 117.00, with a sensitivity of 71.4% and a specificity of 100.0%. The best cut-off value of ROMA score was 46.91, with a sensitivity of 67.9% and a specificity of 94.4%. The best cut-off value of SUVmax was 7.10, with a sensitivity of 85.7% and a specificity of 100.0%.

**Table 1:** Patients’ Characteristics. EOC, Epithelial Ovarian Cancer; CA125, cancer antigen 125; HE4, human epididymis protein, ROMA risk of ovarian malignancy algorithm; SD, standard deviation; SUVmax, maximum standardized uptake value

	Benign (n = 18)	EOC (n = 28)	P value
Age (years) mean±SD	53.9±13.7 (range 22-87)	54.0±18.0 (range 24-86)	0.8131
Menopausal status			
Premenopausal	7 (38.9)	13 (46.4)	
Postmenopausal	11 (61.1)	15 (53.6)	
Stage			
Early stage (I/II)		11 (39.3)	
Advanced stage (III/IV)		17 (60.7)	
CA125 (U/mL)	45.7±67.3	659.5±1114.2	0.0001
HE4 (pmol/L)	54.5±21.4	432.0±473.2	<0.0001
ROMA (%)	17.1±16.6	64.3±36.2	0.0001
SUVmax	2.8±1.7	11.2±4.5	<0.0001

**Table 2:** Histopathological Result of Pelvic Masses

Histopathological results	Number (%)
Epithelial ovarian cancer	28
Serous carcinoma	13 (46.5)
Mucinous carcinoma	4 (14.3)
Endometrioid carcinoma	4 (14.3)
Clear cell carcinoma	2 (7.1)
Seromucinous carcinoma	3 (10.7)
Poorly differentiated carcinoma	2 (7.1)
Benign tumor	18
Serous cystadenoma	1 (5.5)
Mucinous cystadenoma	6 (33.3)
Endometriotic cyst	3 (16.7)
Fibroma	5 (27.8)
Uterine myoma	2 (11.1)
Struma ovarii	1 (5.6)

## Discussion

Early detection and treatment of ovarian cancer may result in a good prognosis; therefore, several tumor markers, such as CA125, HE4, and ROMA score, are used for the early detection of ovarian cancer. The ROMA score, which is the combination of CA125, HE4, and the menopausal status of patients, has recently been used for diagnosing and assessing the prognosis of ovarian cancer (Moore et al., 2009). The effectiveness of F-18 FDG PET/CT for differential diagnosis, staging, and therapy monitoring of ovarian cancer is well known (Zytoon et al., 2013; Rubello et al., 2018; Kemppainen et al., 2019).

In this study, we aimed to compare the diagnostic performance of F-18 FDG PET/CT and the ROMA score. Our study shows that the SUVmax was superior to the ROMA score in distinguishing epithelial ovarian cancer from benign pelvic tumors.

Several studies have reported that the diagnostic performances of HE4 and ROMA score were higher than that of CA125 (Molina et al., 2011; Wei et al., 2016). Some studies have reported that HE4 and ROMA score does not improve the detection rate of ovarian cancer as compared with that of CA125 (Van Gorp et al., 2011). Moreover, several studies have reported that the diagnostic performance of CA125 is higher than that of HE4 in postmenopausal groups (Van Gorp et al., 2011; Romagnolo et al., 2016). In this study, there was no significant difference in the diagnostic performances of CA125, HE4, and ROMA score in all patients.

Previous meta-analyses reported that in early stage ovarian cancer, diagnostic performances of CA125, HE4, and ROMA score were relatively lower than those in advanced stage ovarian cancer (Li et al., 2012; Dayyani et al., 2016). Our study also yielded similar results. Several factors could affect the levels of serum CA125 and HE4. Serum CA125 levels show differences based on the histologic subtype of epithelial ovarian cancers. Serum CA125 levels are high in serous carcinomas but low in mucinous and clear cell carcinomas (Lu et al., 2004; Kobel et al., 2008). CA125 can also be elevated in other cancers and inflammatory or benign gynecological diseases (Sjovall et al., 2002; Moore et al., 2012). HE4 increases steadily with age (Urban et al., 2012). Smoking could elevate serum HE4 levels (Urban et al., 2011; Ferraro et al., 2015).

Previous meta-analyses reported that the diagnostic accuracy of ROMA score in early ovarian cancer was superior to that of CA125 and HE4 (Dayyani et al., 2016). However, in our study, there were no significant differences seen in the diagnostic accuracies of CA125, HE4, and ROMA.

In this study, the diagnostic accuracy of SUVmax was significantly higher than that of CA125 and ROMA score in early ovarian cancer patients. F-18 FDG PET/CT is known to be useful in staging and assessing the prognosis of ovarian cancer. SUVmax is known to exhibit a good diagnostic performance (Zytoon et al., 2013; Rubello et al., 2018; Kemppainen et al., 2019). In this study, SUVmax showed the highest diagnostic accuracy for distinguishing epithelial ovarian cancers and benign pelvic tumors. The best cut-off value of SUVmax was 7.10, with a sensitivity of 85.7% and a specificity of 100%.

Although F-18 FDG PET/CT is expensive as compared to serum tumor markers, SUVmax using F-18 FDG PET/CT showed a high diagnostic accuracy for differentiating epithelial ovarian cancer from benign pelvic tumors. F-18 FDG PET/CT is also useful in staging, especially for assessing metastases (Signorelli et al., 2013; Zytoon et al., 2013; Kemppainen et al., 2019). Metabolic parameters such as metabolic tumor volume and total lesion glycolysis could be used to predict recurrence and to assess the prognosis (Chung et al., 2012; Han et al., 2018). F-18 FDG PET/CT can be a useful modality for the assessment of pelvic masses, including early stage ovarian cancer.

Our study has certain limitations. First, F-18 FDG PET/CT was conducted in patients with pelvic tumors clinically suspected for malignancy. Thus, patients with an early stage ovarian cancer could not be sufficiently included. Second, the number of patients in the subgroup analysis was low because of the low prevalence of ovarian cancers. Further investigations with a larger number of patients are warranted.

In conclusion, SUVmax using F-18 FDG PET/CT showed a high diagnostic accuracy for differentiating epithelial ovarian cancer from benign pelvic tumors, including early stage ovarian cancer. F-18 FDG PET/CT can be a useful modality for the assessment of pelvic mass.

## Author Contribution Statement

SS performed the analysis and wrote this paper. JS helped to collect data. KB, DH and JM contributed to data collection and analysis tool. SM conceived of presented idea and supervised the finding of this work. All authors discussed the results and contributed to final manuscript.
